# Neural Oscillations Associated With Auditory Duration Maintenance in Working Memory in Tasks With Controlled Difficulty

**DOI:** 10.3389/fpsyg.2020.545935

**Published:** 2020-09-03

**Authors:** Xiaolin Yu, Youguo Chen, Ting Luo, Xiting Huang

**Affiliations:** ^1^School of Education Science, Jiangsu Normal University, Xuzhou, China; ^2^Faculty of Psychology, Southwest University, Chongqing, China

**Keywords:** temporal information, duration, working memory, neural oscillation, task difficulty

## Abstract

The neural representation of the external events duration in working memory (WM) remains to be understood. It has been reported that there were different neural representations below and above 3 s/2 s for visual/auditory duration, respectively. However, these studies had limitations in experimental design, i.e., the interference of task difficulty was not assessed. Consequently, the results of these studies require verification. In the present study, we eliminated these limitations using an exploratory experiment in which the probe stimulus conditions were reset, while the other settings remained similar to those used in previous studies. In the exploratory experiment, we found that accuracy and reaction times were comparable among all the four duration conditions, suggesting that task difficulty was accurately matched. In the formal experiment, theta and alpha oscillations were examined using electroencephalogram recordings during the maintenance of the auditory duration in working memory, after removing the interference of task difficulty. Electroencephalogram results indicated that there were no significant differences in theta band power among different length of durations retained in working memory, whereas the alpha band power was significantly lower in the 3-s and 4-s duration conditions than in the 1-s and 2-s conditions. The findings suggest that different internal representations of auditory durations above and below the 2-s threshold are maintained in working memory. Also, our study provides evidence that the duration representation segmentation is associated with the length of the auditory duration retained in working memory, but not with task difficulty.

## Introduction

Although no particular sensory organ specifically perceives time, researchers have tried to understand how time is represented and processed in the brain. Studies have suggested that the internal representation of the duration of external events must be maintained in working memory (WM) before entering long-term reference memory. Additionally, after it is retrieved from long-term memory, the representation of the reference duration can be stored temporally in the WM (Gibbon, 1977; Allan, 1998; Coull et al., 2008). This indicates that WM is essential for processing information related to time. However, little is known regarding temporal information compared with other types of stimuli that can be stored in WM, such as locations, numbers, letters and faces.

Recently, researchers have adopted electroencephalogram (EEG) recordings and the matching-to-sample task to investigate neural oscillations during the maintenance of visual (Chen et al., 2015) and auditory durations (Yu et al., 2017) in WM. Results showed that duration maintenance in WM was associated with alpha (8–12 Hz) activity, especially the significant alpha band power difference between short and long duration retained in WM. Additionally, the findings have also indicated that there is sensory modality difference in the critical threshold point. The alpha activities increased significantly during the visual duration that was maintained in WM for no more than 3 s, while decreased once the duration exceeded 3 s (Chen et al., 2015). Conversely, the critical threshold point was 2 s for auditory duration (Yu et al., 2017). This can be explained by previous findings that auditory signals were often considered as longer than visual signals for a given duration (Goldstone and Lhamon, 1974; Wearden et al., 1998; Penney et al., 2000; Wearden et al., 2006; Droit-Volet et al., 2007).

Some studies have suggested that alpha oscillations are related to the successful maintenance of item information (Jensen and Tesche, 2002; Palva and Palva, 2007; Hsieh et al., 2011; Johnson et al., 2011). Hsieh et al. (2011), for example, found that the posterior alpha oscillations were increased during item maintenance, and primarily in high performers in WM tasks. Additionally, the findings from Chen et al. (2015) and Yu et al. (2017) also emphasized the role of the alpha band in duration maintenance in WM. Therefore, WM can only successfully maintain the short durations within a certain threshold based on the limited capacity of WM. However, WM cannot maintain short durations beyond this threshold, which is manifested as the decrease of alpha activities. This viewpoint is attributed to previous studies of Fraisse (1984) and Pöppel (1997). Fraisse (1984) suggested that perception of duration below the threshold (about 3 s) is based on the subjective present. The sensory inputs are integrated by a low-frequency binding mechanism into a temporal gestalt or coherent experience (Pöppel, 1997). Durations above the threshold can no longer be perceived as a unit because of disintegration, and estimation of longer durations are based on memory and cognitive reconstruction (Fraisse, 1984). This was supported by an event-related potential study (Elbert et al., 1991) and a functional magnetic resonance imaging study (Morillon et al., 2009). Furthermore, the findings from Chen et al. (2015) and Yu et al. (2017) also provided electrophysiological evidence for the different internal representations below and above 2–3 s from different sensory modalities.

Nevertheless, the primary limitation in the experimental design of the existing studies was the lacking in consideration of task difficulty. The behavioral results of two studies showed that the accuracy of duration judgments for visual (Chen et al., 2015) and, especially, for auditory duration (Yu et al., 2017) decreased from the 1-s condition to the 4-s condition, which indicated that the task difficulty was increasing. Previous studies indicated that the alpha activity was lower in difficult task conditions compared with the easy ones (Glass, 1966; Gundel and Wilson, 1992; Gevins et al., 1997). Therefore, it is possible that the decrease in alpha activity for the longer-duration could merely reflect increased task difficulty, i.e., it may be an unspecific effect not related to the length of the duration. The reliability of existing findings needs further confirmation. The primary limitation remains to be the experimental design; specifically, both studies adopted matching-to-sample task, which can separate the maintenance stage from the encoding and decision stages of duration processing. Participants first saw/heard a sample duration (the encoding phase) and then maintained it in WM during the subsequent 3-s interval (the delay/maintenance phase) until the probe duration emerged. At last, they compared the probe duration with the sample one (the decision phase). However, the sample and probe stimuli were presented randomly for 1, 2, 3, or 4 s, which led to a mismatch in reactions among the four sample duration conditions. That is, there were two types of judgments (“equal to” and “longer/shorter than”) for the probe stimulus when the sample stimulus was 1 and 4 s, whereas there were three types of judgments (“equal to,” “longer than,” and “shorter than”) when the sample stimulus was 2 and 3 s. Furthermore, it is more difficult to discriminate the same difference value (e.g., Δ*I* = 1 s) when the sample stimulus is greater (e.g., sample-probe: 4–3 s vs. 1–2 s) based on Weber’s law. All these may contribute to the lower accuracy of increasing sample durations, and it is necessary to retest after controlling the task difficulty.

In the present study, we used electroencephalogram recordings and the modified matching-to-sample task to examine whether there were separate internal representations below and above the 2-s threshold during the maintenance of the auditory duration in WM after eliminating the interference of task difficulty. Specifically, the control of task difficulty in the present study particularly refers to the anticipated difficulty of duration maintenance, i.e., the anticipation of the subsequent task’s difficulty. Anticipated difficulty can influence the adjustment of the information-processing system (Ullsperger et al., 1987) and the allocation of the necessary attentional and processing resources (Wilson et al., 1998). Moreover, anticipated difficulty may also cause different motivational arousal, which could facilitate or interfere with the retention of information (Hill et al., 1985). Therefore, after matching the anticipated difficulty among different duration conditions, we expected the theta power to not be associated with the length of duration retained in WM, which would be consistent with the findings in previous studies (Chen et al., 2015; Yu et al., 2017). As for the alpha band, which was our focus, if differences in alpha activities below and above the 2-s threshold were observed, then it would imply that the changes of alpha activities are primarily dependent on the length of the auditory duration retained in WM, but not the task difficulty. Otherwise, it would manifest the correlation between duration segmentation and task difficulty, which warrants further research.

## Materials and Methods

### Participants

Sixty undergraduate students (21 male and 39 female, 20.267 ± 1.656 years old) participated in the exploratory experiment, and an additional 26 undergraduate students (10 male and 16 female, 20.308 ± 1.871 years old) participated in the formal experiment. All right-handed participants had normal or corrected-to-normal vision and normal hearing. They were paid for their participation after they provided informed written consent. This study was approved by the local institutional review board of Southwest University. All experiments performed were compliant with the ethical standards of the Declaration of Helsinki (World Medical Association, 2013).

### Experimental Material and Apparatus

Binaurally presented pure tones of 1,000 Hz were used as the auditory stimuli and presented through Sennheiser stereo earphones. The sound decibel was adjusted to ∼60 dB, which was comfortable for the subjects. The duration of the sample stimulus was 1, 2, 3, or 4 s, while the durations of the probe stimuli were adjusted in the exploratory experiment based on Weber’s law to achieve the same task difficulty among the four duration conditions, i.e., the duration of the probe stimulus was 0.5, 1, or 1.5 s when the sample stimulus was 1 s; the duration of the probe stimulus was 1, 2, or 3 s when the sample stimulus was 2 s; the duration of the probe stimulus was 1.5, 3, or 4.5 s when the sample stimulus was 3 s; and the duration of the probe stimulus was 2, 4, or 6 s when the sample stimulus was 4 s. The response signal was a 2-cm white question mark. The computer screen was positioned approximately at 75 cm from the participants’ eyes. The refresh rate of the monitor was 85 Hz.

### Procedure

**FIGURE 1:**
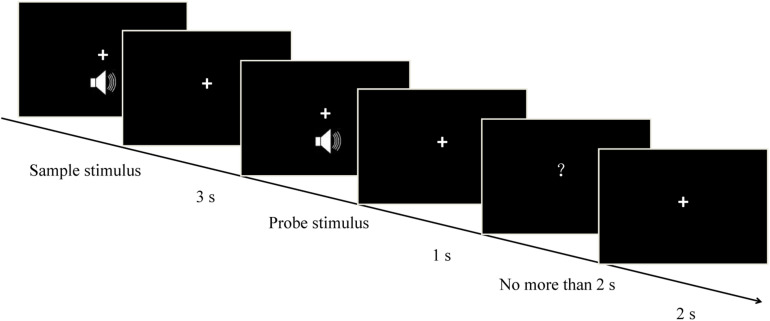
Schematic representation of each individual trial. Each trial started with a pure tone (sample stimulus), which was randomly presented for 1, 2, 3, or 4 s. After a 3-s interval (the delay/maintenance phase), a second pure tone (probe stimulus) was presented randomly for one of three possible durations depending on the sample duration (i.e., sample – probe: 1–0.5 s, 1 s, 1.5 s; 2–1 s, 2 s, 3 s; 3–1.5 s, 3 s, 4.5 s; 4–2 s, 4 s, 6 s). Participants estimated whether the duration of the probe stimulus was shorter, equal to, or longer than that of the sample one, and correspondingly pressed “1,” “2,” or “3,” respectively.

The temporal version of the matching-to-sample task was used in the present study (Figure 1). The procedures of the exploratory experiment and the formal experiment were identical, each consisting of seven blocks. Participants heard two successive pure tones (the sample stimulus and the probe stimulus) with a 3-s interval (the delay/maintenance phase) between them. The sample stimulus was randomly presented for 1, 2, 3, or 4 s, and each one corresponded to three randomly presented probe stimuli, resulting in a total of 12 duration conditions (i.e., sample-probe: 1–0.5 s, 1 s, 1.5 s; 2-1 s, 2 s, 3 s; 3-1.5 s, 3 s, 4.5 s; 4–2 s, 4 s, 6 s). After a 1-s interval, a question mark (response signal) was presented on the screen. This signal elapsed after a key press, or after 2 s. Participants were asked to compare the durations of the probe stimulus and the sample stimulus. They had to ascertain whether the duration of the probe stimulus was shorter, equal to, or longer than that of the sample stimulus, and press “1,” “2,” or “3,” respectively. Half the participants were instructed to respond using their left hand, and the other half were instructed to respond using their right hand. The intertrial interval was 2 s. Participants were monitored via video camera to make sure that they did not close their eyes during auditory stimulation to eliminate possible effects of participants’ eye closure on neural oscillations. There were 84 trials for every sample stimulus, and 336 trials in total. In this experiment, the time taken for the participants to respond and the accuracy of responses were measured.

### Electrophysiological Recording

The EEG was conducted using 64 Ag/AgCl scalp electrodes mounted in an elastic cap (Brain Products GmbH, Herrsching, Germany). The positioning of the electrodes conformed to the extended 10/20 system, and additional electrodes were placed on the mastoids. The horizontal and vertical electrooculogram (EOG) were recorded using electrodes positioned at the ocular canthi and below the right eye, respectively. The EEG and EOG were digitized at a rate of 500 Hz with an amplifier bandpass of 0.01–100 Hz, including a 50-Hz notch filter. Impedances for all electrodes were maintained below 5 kΩ.

### EEG Analysis

Offline EEG data were processed using EEGLAB (Delorme and Makeig, 2004) for MATLAB (The MathWorks, Natick, MA, United States). Recordings were re-referenced to the average of the left and right mastoids, and high-pass filtered at 0.5 Hz (Hsieh et al., 2011; Roberts et al., 2013; Chen et al., 2015; Yu et al., 2017). The 9-s time windows (1 s pre-stimulus and 8 s post-stimulus, around the time of sample stimulus onset) were epoched using the raw EEG data. All epochs were baseline-corrected by subtracting the mean voltage before the sample stimulus. Subsequently, epochs with amplitude exceeding ±100 μV were automatically marked and manually confirmed and removed through visual inspection. Artifacts caused by eye blinks and movements were removed using an independent component analysis using the scalp map and activity profile of the participant (Jung et al., 2000a,b). Thus, 95.20% of trials remained and the number of remaining trials was not significantly different among the four duration conditions of the sample stimulus [*F*(3, 75) = 0.517, *p* > 0.05, η*_*p*_*^2^ = 0.020].

The time–frequency analysis was performed using Hanning-windowed sinusoidal wavelets, for which the cycle number linearly increases with frequency, from 3 cycles for 3 Hz to approximately 15 cycles for 30 Hz (Makeig et al., 2004; Chen et al., 2015; Yu et al., 2017). Using this method, the trade-off between temporal resolution at lower frequencies and stability at higher frequencies leads to optimization (Makeig et al., 2004). We adopted the event-related spectral perturbation (ERSP) index (Makeig, 1993) to compute the changes in the event-related spectral power response (in dB), which was based on single trials. The baseline interval of the decibel transformation was 0.4–0.1 s before the sample stimulus.

**FIGURE 2:**
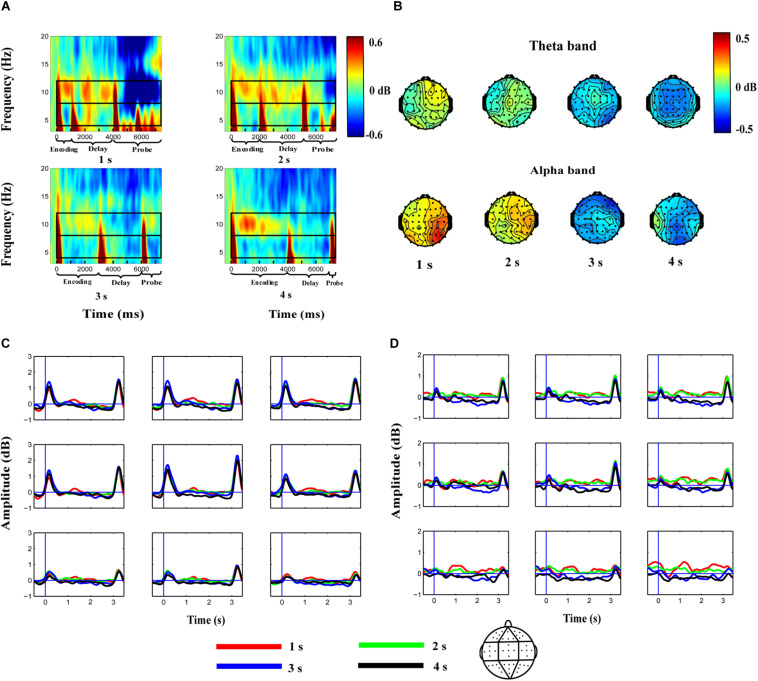
Average EEG spectrum power for the whole epoch **(A)**, topographies of theta and alpha activity in the delay phase **(B)**, and average theta **(C)** and alpha **(D)** band powers during the maintenance of the auditory duration in WM in the 1, 2, 3, and 4-s duration conditions. **(A)** Decibel-transformed was relative to the baseline interval (−0.4 to −0.1 s) before the sample stimulus. Theta oscillations are enhanced at the onset and offset of stimuli, whereas alpha oscillations are enhanced during the encoding, especially the delay phase. **(B)** Topographies of theta and alpha activity in the 1, 2, 3, and 4-s conditions during auditory duration maintenance in WM. Alpha band powers mainly activated in the posterior from the interval of 1–3 s after the onset of delay. **(C,D)** Steady theta (4–8 Hz) and alpha (8–12 Hz) band activities are elicited during WM maintenance after averaging across frequency and are separately plotted for each of the nine analyzed electrode clusters. Zero of the x-axis represents the onset of the delay phase. Red, green, blue, and black curves denote the 1, 2, 3, and 4-s auditory duration conditions respectively.

Using the method described in previous studies (Roberts et al., 2013; Yu et al., 2017), electrodes were grouped into nine clusters: the left-frontal cluster (Fp1, AF3, AF7, F3, F5); the middle-frontal cluster (Fpz, F1, Fz, F2); the right-frontal cluster (Fp2, AF4, AF8, F4, F6); the left-central cluster (FC5, C3, C5, T7, CP5); the middle-central cluster (FCz, Cz, C1, C2, CPz); the right-central cluster (FC6, C4, C6, T8, CP6); the left-posterior cluster (P3, P5, P7, PO3, O1); the middle-posterior cluster (P1, P2, Pz, POz, Oz); and the right-posterior cluster (P4, P6, P8, PO4, O2). Figure 2A shows the average ERSP of the nine clusters for the 1-s, 2-s, 3-s, and 4-s duration conditions during the encoding, delay, and probe phases of the sample condition, relative to the baseline. The topographies of theta and alpha activities in the delay phase are shown in Figure 2B. Additionally, theta (Figure 2C) and alpha (Figure 2D) band powers were extracted after averaging across frequencies to identify the temporal dynamics of the oscillations during WM maintenance. Zero represented the onset of delay in this new coordinate system.

A complementary analysis was also conducted for the 0.5–0.1 s time window before the onset of delay. This analysis was performed to examine whether the subsequent statistical analyses had been contaminated owing to different time intervals. A Greenhouse–Geisser correction was adopted for the violation of sphericity assumption (Greenhouse and Geisser, 1959).

## Results

### Accuracy and Reaction Time in the Exploratory Experiment

**FIGURE 3:**
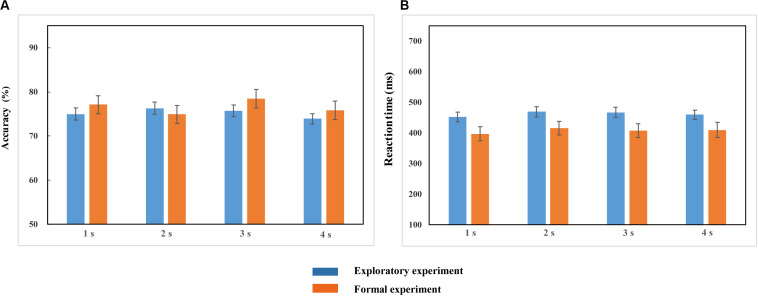
Accuracy **(A)** and Reaction time **(B)** in the 1, 2, 3, and 4-s duration conditions in the exploratory experiment and formal experiment. Blue and red bars represent the accuracy **(A)** and reaction time **(B)** of four duration conditions in the exploratory experiment and the formal experiment, respectively. Error bars represent the SEM across observers.

Repeated-measures ANOVA conducted on the accuracy (ACC) and reaction time (RT) demonstrated no significant effects of duration {ACC: [*F*(2.248, 132.623) = 1.972, *p* > 0.05, η*_*p*_*^2^ = 0.032]; RT: [*F*(2.453, 144.752) = 2.087, *p* > 0.05, η*_*p*_*^2^ = 0.034]; Figure 3}. This suggests that the duration settings of the probe stimulus achieved the equal difficulty among all different duration conditions (Table 1), which can be further applied to the formal experiments.

**TABLE 1:** Mean accuracies and reaction times (standard error) in the exploratory experiment and formal experiment.

		**1 s**	**2 s**	**3 s**	**4 s**
Exploratory experiment	Accuracy	0.750(0.014)	0.763(0.014)	0.757(0.013)	0.739(0.012)
	Reaction time	453.092 (15.865)	469.564 (16.866)	467.046 (16.514)	459.787 (15.073)
Formal experiment	Accuracy	0.771 (0.020)	0.749 (0.020)	0.785 (0.021)	0.758 (0.021)
	Reaction time	397.099 (23.068)	415.301 (22.193)	407.530 (22.048)	409.925 (24.755)

### Accuracy and Reaction Time in the Formal Experiment

Repeated-measures ANOVA conducted on the accuracy and reaction time revealed that there were no significant differences in accuracy [*F*(2.474, 61.843) = 1.889, *p* > 0.05, η*_*p*_*^2^ = 0.070] and reaction time [*F*(2.281, 57.031) = 1.476, *p* > 0.05, η*_*p*_*^2^ = 0.056] among the four duration conditions (Figure 3). It proved that the task difficulty was controlled among different duration conditions.

### EEG Characteristics in the Formal Experiment

Alpha band power in the interval of 0.5–0.1 s prior to the onset of delay analyzed using the repeated-measures ANOVA revealed no significant effect of duration or duration × region interaction (all *p* > 0.05). However, we observed a significant main effect of duration [*F*(2.164, 54.097) = 5.749, *p* < 0.01, η*_*p*_*^2^ = 0.187] on the alpha band power in the interval of 1–3 s after the onset of delay. *Post-hoc* comparisons indicated that the difference in the alpha power between the 1-s (0.217 ± 0.158 dB) and 2-s (0.150 ± 0.200 dB) conditions was not significant [*t*(25) = 0.610, *p* > 0.05]. Similarly, the difference between the 3-s (−0.259 ± 0.228 dB) and 4-s (−0.231 ± 0.172 dB) conditions was not significant [*t*(25) = −0.181, *p* > 0.05]. However, the alpha band power in the 1-s and 2-s conditions was higher than that in the 3-s and 4-s conditions [*t*(25) = 2.129–4.019, all *p* < 0.05]. The main effect of region [*F*(3.582, 89.544) = 0.572, *p* > 0.05, η*_*p*_*^2^ = 0.022] and the duration × region interaction [*F*(7.383, 184.581) = 1.529, *p* > 0.05, η*_*p*_*^2^ = 0.058] were not significant (Figure 2D).

Theta band power in the interval of 0.5–0.1 s prior to the onset of delay analyzed by a repeated-measures ANOVA did not reveal a significant effect of duration or a duration × region interaction (all *p* > 0.05). A similar result was observed for the theta band power in the interval of 1–3 s after the onset of delay, i.e., there was no significant effect of duration [*F*(3, 75) = 1.655, *p* > 0.05, η*_*p*_*^2^ = 0.062], region [*F*(1.818, 45.446) = 0.341, *p* > 0.05, η*_*p*_*^2^ = 0.013], or a duration × region interaction [*F*(6.389, 159.728) = 0.700, *p* > 0.05, η*_*p*_*^2^ = 0.027] (Figure 2C).

## Discussion

To better understand neural oscillations during the maintenance of the auditory duration in WM, we eliminated the limitations existent in previous studies by matching the task difficulty of every duration condition in the present study. Subsequently, we used a modified matching-to-sample task to investigate whether there were separate internal representations below and above the 2-s threshold during the auditory duration maintenance in WM after eliminating the interference of task difficulty. EEG results indicated that the theta band power during the delay phase was not significantly modulated by the length of the auditory duration, which confirmed our hypothesis that the theta power would not associate with the length of duration retained in WM. More importantly, the alpha band power was significantly lower in the 3-s and 4-s duration conditions than in the 1-s and 2-s conditions, which showed that the changes of alpha activities were primarily dependent on the length of the auditory duration retained in WM but not on the task difficulty.

Influenced by the characteristics of duration information, there were different time intervals between the baseline and delay phase among the 1-s, 2-s, 3-s, and 4-s duration conditions. Thus, the alpha band power during the interval of 0.5–0.1 s prior to the onset of the delay phase might have been affected by this factor. Nevertheless, no significant main effect of duration or duration × region interaction was observed for the theta and alpha band power during the interval of 0.5–0.1 s prior to the delay phase, which indicated that the factor was not relevant. Additionally, different neural systems are recruited for the measuring of sub-second and supra-second intervals, which is supported by previous neuroimaging studies (Jahanshahi et al., 2006; Morillon et al., 2009; Wiener et al., 2010; Hayashi et al., 2014). However, there are two opposite hypotheses concerning the temporal cognition segmentation in the seconds range: segmentation and non-segmentation hypotheses. The segmentation hypothesis holds that there may be distinct processing mechanism and representation of different length of time (Fraisse, 1984; Pöppel, 1997; Kagerer et al., 2002; Wittmann et al., 2007), while a single mechanism is suggested to explain the whole range of possible durations by non-segmentation hypothesis (Church, 1984; Fortin and Couture, 2002; Gibbons and Rammsayer, 2004). Our study provides electrophysiological evidence for the segmentation hypothesis, which emphasizes the distinct neural representations below and above 2 s influenced by the length of duration.

The speculative explanation for the change of representations when the durations exceed 2–3 s was based on the subjective present or “states of being conscious” by Fraisse (1984) and Pöppel (1997). We can also show the mechanism of segmentation through the functional role of the alpha band frequencies. Although task difficulty was not controlled in the previous two studies mentioned (Chen et al., 2015; Yu et al., 2017), they conducted the correlation analysis between the alpha band power and accuracy of duration judgments which supported the important role of alpha oscillations in the successful maintenance of duration, showing that WM can only successfully maintain the short durations within 2–3 s accompanied by the increase of alpha activities. Equally, longer durations cannot be maintained in WM as shown by the decrease of alpha activities. Although the role of alpha oscillation was not the focus of our study, the consistent results with the previous study (Yu et al., 2017) regarding the critical threshold point, which was the same, and the topography and encoding-related activity, which were equivalent (although they did not control for task difficulty), not only demonstrate the reliability of our study, but also provide the same explanation for the segmentation of duration representation.

Lastly, there are some limitations in our study. Task difficulty is primarily reflected in the comparison accuracy of sample and probe durations based on the matching-to-sample task, so the experimental design was modified in our study to achieve the non-significant task difficulty among the four duration conditions according to Weber’s law. However, it is not suitable to investigate the relationship between alpha effect in the delay phase and the WM performance after controlling the accuracy of different conditions, which was not the focus of our study. Although the accuracy of time comparison is often considered as an index of WM performance, it is inappropriate to predict it only by using alpha activities in WM maintenance. Since the accuracy of matching-to-sample task is combined, it is affected by encoding, maintenance and decision processes for temporal information. A specific approach is needed to investigate the role of alpha band in duration WM performance in future studies. In addition, the present study mainly focuses on the implicit anticipated difficulty. In a further study, we could convert it to the explicit one to investigate the effect of anticipated difficulty on duration maintenance. For example, weak distracters (i.e., stimuli that are different from duration) or strong distracters (i.e., stimuli that are similar to duration) could be presented during WM maintenance in a modified version of the Sternberg paradigm presented in the study of Bonnefond and Jensen (2012). The details still require further discussion. Finally, the use of EEG by current studies to explore the neural oscillations in duration maintenance in WM remains a preliminary assessment. This calls for further studies using various advanced techniques (e.g., functional magnetic resonance imaging with high spatial resolution) to elucidate the precise neural mechanism and localization.

## Data Availability Statement

The raw data supporting the conclusions of this article will be made available by the authors, without undue reservation, to any qualified researcher.

## Ethics Statement

This study was reviewed and approved by the local institutional review board of Southwest University. All participants provided their written informed consent to participate in this study.

## Author Contributions

XY, YC, and XH developed the experimental idea. XY and YC designed the experiments. XY collected the data and analyzed with TL. All authors contributed to the article and approved the submitted version.

## Conflict of Interest

The authors declare that the research was conducted in the absence of any commercial or financial relationships that could be construed as a potential conflict of interest.
